# Chemogenetic modulation of CRF neurons in the BNST compensates for phenotypic behavioral differences in fear extinction learning of 5-HT2C receptor mutant mice

**DOI:** 10.1038/s41398-025-03799-1

**Published:** 2026-01-10

**Authors:** Hannah Schulte, Hanna Böke, Patricia Lössl, Maria Worm, Ida Siveke, Stefan Herlitze, Katharina Spoida

**Affiliations:** 1 Department of Zoology and Neurobiology, ND7/31, Ruhr-University Bochum, Universitätsstr. 150, D-44780 Bochum, Germany; 2 Bridge Institute of Experimental Tumor Therapy, University Hospital Essen, University of Duisburg-Essen, Essen, 45147 Germany

**Keywords:** Psychiatric disorders, Learning and memory

## Abstract

Psychopharmacotherapy is often used to treat anxiety- and stress-associated psychiatric disorders, including post-traumatic stress disorder (PTSD). Adjunctive therapy is most typically used with medications that influence serotonin balance, such as selective serotonin reuptake inhibitors (SSRIs). Contrary to expectations, SSRIs show an anxiety-increasing effect during the initial treatment phase. Among the 14 different serotonin receptor subtypes, pharmacological studies have demonstrated that 5-HT2C receptors (5-HT2CRs) in the bed nucleus of the stria terminalis (BNST) play a significant role in the anxiogenic effect of acute SSRI treatment. Although numerous studies have confirmed the role of the 5-HT2CR in anxiety behavior, little is known about its involvement in learned fear and fear extinction. In particular, fear extinction is considered a central neural mechanism in the treatment of PTSD patients. Recent results from 5-HT2CR knockout mice (2CKO) revealed that global loss of 5-HT2CRs enhances fear extinction, without affecting fear acquisition. Here, we implemented a chemogenetic approach to examine the neuronal substrate which underlies this extinction-enhancing effect in 2CKO mice. DREADD-activation of BNST^CRF^ neurons promotes fear extinction in 5-HT2CR WT mice, whereas DREADD-inactivation of BNST^CRF^ neurons impairs fear extinction in 2CKO mice. Thus, using activating and inactivating DREADDs, we were able to bidirectionally modulate fear extinction. These findings provide a possible explanation for the fear extinction-enhancing effect in 2CKO mice with relevance for the treatment of PTSD patients.

## Introduction

Fear and anxiety are closely related emotions. Anxiety is characterized by persistent, excessive worries that are pursued even in the absence of an immediate threat. In contrast, fear is a natural response to a specific, known danger [1]. Although anxiety and fear are fundamentally different emotional states, they may share common neural and behavioral mechanisms [2]. Dysfunctions in the underlying neural networks may lead to anxiety, fear, and stress-related disorders, such as post-traumatic stress disorder (PTSD) [3, 4].

From a neurobiological perspective, the development of PTSD is linked to classical fear conditioning, while its persistence is attributed to a malfunction in extinction learning [3, 4]. Individuals with PTSD often struggle to extinguish the learned association between a neutral environmental stimulus and the fear response, which can impair the efficacy of therapeutic interventions.

In laboratory settings, fear conditioning (FC) and fear extinction (FE) paradigms are commonly used experimental approaches to study the neurobiological features of aversive learning that contribute to PTSD [5]. FC involves repeatedly pairing a neutral, nonthreatening stimulus, like a tone (conditioned stimulus, CS), with an aversive stimulus, such as a mild foot shock (unconditioned stimulus, US), until the animal begins to display a fear response not only to the shock but also to the tone alone (conditioned response, CR) [5, 6]. On the other hand, extinction learning is an important biological adaptation mechanism that allows old behavior patterns to be unlearned when they are linked to invalid information. It is important to note that extinction learning suppresses the originally acquired fear association without erasing or overwriting it [7, 8]. Extinction learning is achieved by repeatedly presenting the CS in the absence of the US, reducing the CR, and weakening the previously learned association between a stimulus and a defensive reaction [8].

Studying the cognitive mechanisms behind the formation and extinction of fear contributes to a better understanding of the emergence, maintenance, and treatment of PTSD. In this context, the amygdala is the crucial brain region responsible for forming the association between the signal of an aversive event and the aversive stimulus, giving them emotional significance [9]. Convergent evidence has implicated the amygdala in the extinction of fear [10], while more recent work also points toward a key role for the BNST in threat anticipation and fear modulation [11–16]. The BNST is highly interconnected with the central amygdala (CeA) [17, 18], however, early research on the rodent BNST revealed a functional division between the amygdala and the BNST, supported by human fMRI data. These findings imply that the BNST is implicated in mediating specific fear and anxiety-like behaviors that are communicated without reference to the physical CeA circuit [19]. Within this context, the BNST mediates more diffuse, prolonged fear or unconditioned anxiety states, while the amygdala mediates impending, phasic fear experiences [19]. More recent research, however, suggests that the BNST is also involved in processing phasic, discrete stimuli [11, 13, 20, 21]. Early BNST lesion experiments demonstrated that fear responses were reduced when directly exposed to an aversive stimulus, such as predator scents [22]. Recent electrophysiological recordings in rodents demonstrated enhanced activity of BNST neurons during fear acquisition and CS-dependent fear recall [11, 14, 23, 24]. Another study demonstrated that chemogenetic activation of GABAergic neurons in the BNST during fear conditioning or memory consolidation using a designer receptor exclusively activated by designer drugs (DREADD), which activates the G_q_-pathway, increased CS fear recall without affecting fear expression during conditioning or recall. This implies a modulatory role of the BNST in fear memory formation [11]. Additionally, studies have shown that the BNST is also highly involved in PTSD symptoms like altered hypervigilance, arousal states, and increased sensitization to the environment [19, 25]. Despite the role of the BNST in anxiety behavior, the knowledge about how it affects fear learning,particularly concerning explicit cues and fear extinction, is still emerging.

Serotonin (5-HT) plays a pivotal role in anxiety- and fear-related behaviors through its effects in the BNST [26]. The BNST contains various serotonin receptor subtypes, which are expressed differently across neuron types [27, 28]. Depending on the subtype involved, serotonin can elicit either inhibitory or excitatory neuronal responses [27]. Among the 14 serotonin receptor subtypes, the 5-HT1A and 5-HT2C receptors in the BNST have received particular attention in recent years, as their activation has opposing effects on fear behavior. In the anterodorsal subregion of the BNST (BNSTad), 5-HT1A receptor (5-HT1AR) activation has anxiolytic (anxiety-reducing) effects, while 5-HT2C receptor (5-HT2CR) stimulation increases anxiety [13, 29, 30].

Optogenetic and chemogenetic approaches revealed that 5-HT input to the BNST from the DRN elicits anxiogenic behavior and increased fear learning via 5-HT2CR signaling [13, 31]. Although numerous studies in recent years have examined the role of the BNST in the acquisition, expression, and recurrence of fear, data on fear extinction are completely lacking. In our previous study, we discovered that the BNSTov and the BNSTad play opposing roles in fear extinction, with WT mice showing an extinction-associated reduction in BNSTov activity and an increase in the BNSTad [32]. Optogenetic interventions produced comparable outcomes in anxiety tasks, showing that the BNSTov has an anxiogenic role, whereas the BNSTad has been described as anxiolytic [33]. Similarly, we discovered altered activity in the dorsal BNST in 2CKO mice in an anxiolytic and extinction-supporting direction, even under basal conditions [32]. 2CKO animals had previously been characterized as having an anxiolytic phenotype, with reduced cFos expression in BNST corticotropin-releasing factor (CRF)-expressing neurons in response to anxiety-inducing stimuli [34].

Building on our previous research, we used a chemogenetic approach to investigate the neuronal network in the BNSTad underlying the extinction-enhancing effect in 2CKO mice. Our results revealed that chemogenetic modulation of BNST^CRF^ neurons produces bidirectional effects on the fear extinction phenotype. Activation of BNST^CRF^ neurons using a G_q_-coupled (hM3Dq) DREADD promotes fear extinction in 5-HT2CR WT mice, while inactivation of BNST^CRF^ neurons with a G_i_-coupled (hM4Di) DREADD impairs fear extinction in 2CKO mice.

The findings reported here provide a mechanistic explanation for the extinction-enhancing effect in 2CKO mice. The effect that we observed may be a key mechanism behind SSRI-induced anxiolysis, since the anxiolytic effects of systemic long-term SSRI administration are associated with 5-HT2CR desensitization [35].

## Materials and methods

### Subjects

Adult male mice (9–17 weeks of age) were used for all experiments. To generate a transgenic CRF-ires-Cre/5-HT2CR mouse line, CRF-ires-Cre mice (B6(Cg)-*Crh*^*tm1(cre)Zjh*^/J, stock no. 012704, Jackson Laboratory) were bred with 5-HT2CR knockout (KO) mice (B6.129-Htr2c ^tm1Jul^/J, stock no. 002627, Jackson Laboratory). All mice had either a hemizygous (2CKO) or wild-type (WT) background for the 5-HT2CR allele and were homozygous for the CRF-ires-Cre background. Mice were group-housed (2–5 individuals per cage) with a constant room temperature and a 12 h light/dark cycle while food and water were provided *ad libitum*. All experiments were performed during the light period. The experiments were conducted with the approval of the local ethics committee (Bezirksamt Arnsberg) and the animal care committee of North Rhine-Westphalia (LANUV; Landesamt für Umweltschutz, Naturschutz und Verbraucherschutz Nordrhein-Westfalen, Germany; AZ. 81-02.04.2021.A412). Studies were conducted in compliance with the 2010 European Communities Council Directive (2010/63/EU) for care of laboratory animals and supervised by the animal welfare commission of the Ruhr-University Bochum.

### Viral constructs and Stereotaxic DREADD injections

Double-floxed adeno-associated viruses (AAVs) were used to express Gq-coupled (AAV1.pAAV.hSyn.DIO.hM3D(Gq)-mCherry, Titer: 2.2 × 1013, #44361, Addgene) or Gi-coupled (AAV1.pAAV.hSyn.DIO.hM4D(Gi)-mCherry, Titer: 2.3 × 1013, #44362 Addgene) DREADDs in the BNSTad. For analgesia, subcutaneous injections of buprenorphine (0.1 mg/kg) and carprofen (5 mg/kg) were administered 30 min prior to surgery. The animals were anesthetized with isoflurane (initially at 5% (v/v) and then maintained at 1.5 ± 0.5% (v/v)) and positioned in a stereotaxic frame (Stoelting). The scalp was treated with lidocaine as a local anesthetic. The viral constructs were bilaterally injected into the BNSTad through pressure injection utilizing custom-pulled glass pipettes (tip diameter 5–10 µm). The following stereotactic coordinates relative to bregma were used: AP + 0.26, ML + /− 0.75, DV −4.1 [32]. AAV-injected animals were kept in their home cages for two to three weeks to allow for sufficient virus expression.

### Drug application

Chemogenetic activation within the BNSTad was achieved via intraperitoneal (i.p.) injection of the designer ligand clozapine-N-oxide (CNO; Tocris Bioscience, Cat. No. 6329). CNO was administered at a dose of 1 mg/kg, 40 min prior to the extinction session. Following viral transduction, DREADD-expressing 2CKO and WT mice were randomly assigned to treatment groups. Control groups (WT-Saline and 2CKO-Saline) received an intraperitoneal injection of 0.3 ml of 0.9% saline, also 40 min prior to extinction learning.

To confirm the efficacy of CNO in activating or inhibiting DREADD-expressing neurons, cFos expression was assessed as a marker of neuronal activity. For this, separate groups of mice received either CNO (1 mg/kg, i.p.) or saline (0.3 ml, i.p.) while in their home cages, two hours before perfusion.

### Behavioral testing

**Fig. 1:**
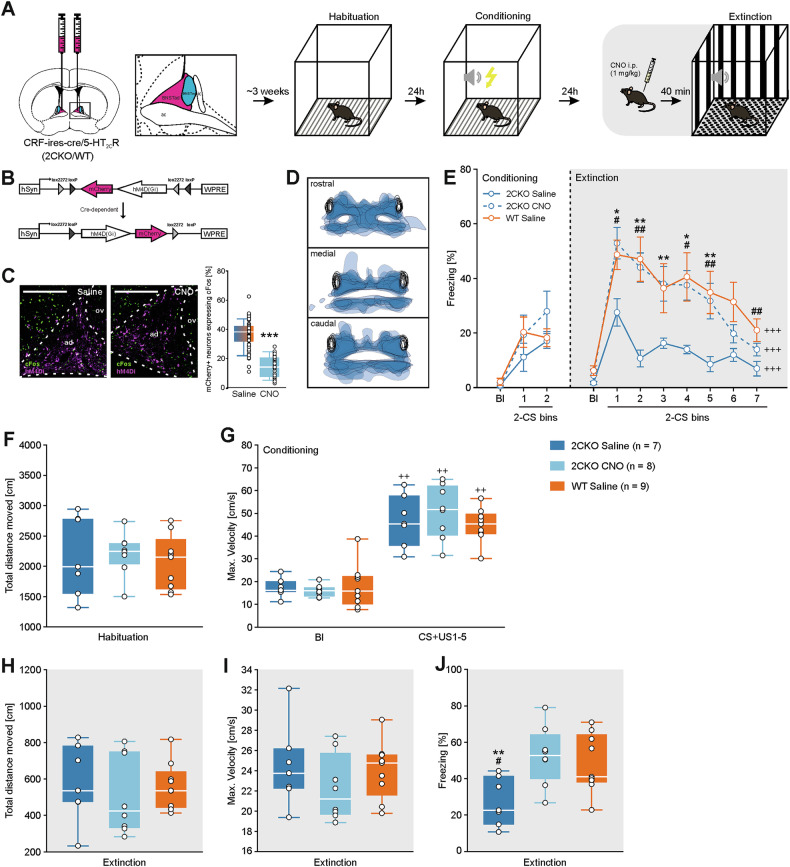
Chemogenetic inactivation of CRF neurons in the BNSTad impairs fear extinction. **A** Schematic representation of the stereotaxic AAV injection encoding the inhibitory hM4D(Gi) DREADD, followed by a three-day fear conditioning and extinction paradigm, using CRF-ires-cre/5-HT_2C_R-KO (2CKO) mice and their wild-type (WT) littermates. DREADD activation was achieved by i.p. injection (CNO; 1 mg/kg) 40 min prior to fear extinction. **B** Illustration of the viral construct encoding hM4Di-mCherry, under control of the Cre/*LoxP* system, driven by human synapsin promoter (hSyn). **C** Representative images showing chemogenetic inactivation of BNST^CRF^ neurons following CNO administration compared to saline-treated controls (scale bar = 250 µm). Images demonstrate a marked decrease in cFos-positive nuclei colocalized with hM3Dq-mCherry in the BNSTad following CNO treatment, confirming effective inactivation of the targeted neuron population (***p < 0.001; Kruskal-Wallis test; Saline: total quantified ROIs: n = 58 derived from 6 animals; CNO: total quantified ROIs: n = 29 derived from 6 animals). **D** Redrawing and overlay of viral spreading within the BNST region. **E** Freezing behavior was analyzed during the baseline period (Bl) and conditioned stimulus (CS) presentation on the days of fear conditioning (day 2) and extinction (day 3). Freezing behavior decreased over time in all groups (^+++^p < 0.001; Friedman’s test; ^+++^p < 0.001; RM-ANOVA).^.^ 2CKO mice showed less freezing behavior during fear extinction compared to WT littermates (^#^p < 0.05; ^##^p < 0.010; Kruskal-Wallis test followed by Dunn’s post-hoc test). Chemogenetic inactivation of BNST^CRF^ neurons enhanced freezing behavior of 2CKO mice (*p < 0.050; **p < 0.010; Kruskal-Wallis test followed by Dunn’s post-hoc test). Data are shown as means ± SEM. **F** Total distance moved during habituation (day 1) did not differ between groups. **G** Maximum velocity increased in all groups during CS and unconditioned stimulus (US) pairings on conditioning day (day 2) compared to the Bl period (^++^p < 0.010; Friedman’s test). **H** The total distance moved during the Bl period of fear extinction did not differ between groups. **I** There were no group variations in maximum velocity during the Bl period of fear extinction. **J** Freezing behavior during fear retrieval (average freezing during the first two CS presentations on fear extinction day; bin1) revealed significant differences between genotypes and treatment groups (^##^p < 0.050; *p < 0.010; two-way ANOVA). For figures F-J) boxes represent the interquartile range (IQR), including the 1^st^ and 3^rd^ quartiles, line within boxes indicates the median and range (whiskers) extend to the upper and lower quartiles within the 1.5 IQR.

**Fig. 2:**
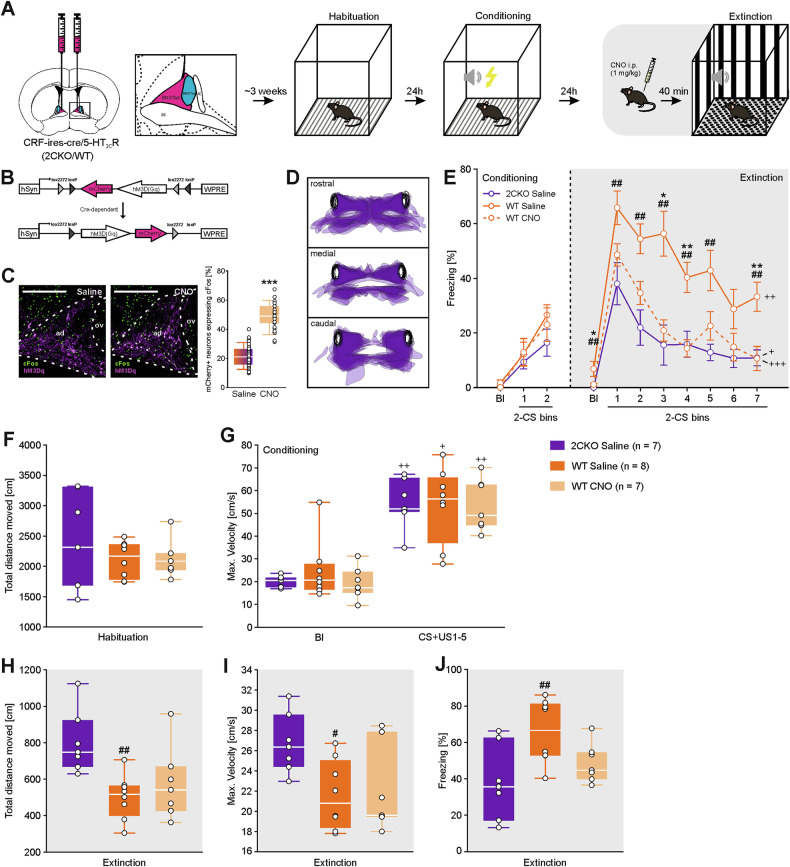
Chemogenetic activation of CRF neurons in the BNSTad facilitates fear extinction. **A** Schematic representation of stereotaxic AAV injection encoding the excitatory hM3D(Gq) DREADD and the subsequent three-day fear conditioning and extinction paradigm, using CRF-ires-cre/5-HT_2C_R-KO (2CKO) mice and their wild-type (WT) littermates. DREADD activation was achieved by i.p. injection (CNO; 1 mg/kg) 40 min prior to fear extinction. **B** Illustration of the viral construct encoding hM3Dq-mCherry, under control of the Cre/*LoxP* system, driven by human synapsin promoter (hSyn). **C** Representative images showing chemogenetic activation of BNST^CRF^ neurons following CNO administration compared to saline-treated controls (scale bar = 250 µm). Images demonstrate a marked increase in cFos-positive nuclei colocalized with hM3Dq-mCherry in the BNSTad following CNO treatment, confirming effective activation of the targeted neuron population (***p < 0.001; Kruskal-Wallis test; Saline: total quantified ROIs: n = 44 derived from 4 animals; CNO: total quantified ROIs: n = 27 derived from 3 animals). **D** Redrawing and overlay of viral spreading within the BNST region. **E** Freezing behavior was analyzed during the baseline period (Bl) and conditioned stimulus (CS) presentation on the days of fear conditioning (day 2) and extinction (day 3). Freezing behavior decreased over time in all groups (^+^p < 0.050; ^++^p < 0.010; ^+++^p < 0.001^;^ Friedman’s test). 2CKO mice showed less freezing behavior on the day of fear extinction compared to WT mice (^##^p < 0.010; Kruskal-Wallis test followed by Dunn’s post-hoc test). Chemogenetic activation of BNSTad CRF neurons reduced freezing behavior of WT mice (*p < 0.050; **p < 0.010; Kruskal-Wallis test followed by Dunn’s post-hoc test). Data are shown as means ± SEM. **F** Total distance moved during habituation (day 1) did not differ between groups. **G** Maximum velocity increased in all groups during CS and unconditioned stimulus (US) pairings on conditioning day (day 2) compared to the Bl period (^+^p < 0.050; ^++^p < 0.010; Friedman’s test). **H** Total distance moved during the Bl of fear extinction revealed a genotype-specific difference (^##^p < 0.010; two-way ANOVA). **I** Maximum velocity during the Bl of fear extinction revealed genotype-specific differences (^#^p < 0.050; two-way ANOVA). **J** Freezing behavior during fear retrieval (average freezing during the first two CS presentations on fear extinction day; bin1) revealed genotype specific differences (^##^p < 0.010; two-way ANOVA). For figures F-J) boxes represent the interquartile range (IQR), including the 1^st^ and 3^rd^ quartiles, line within boxes indicates the median and range (whiskers) extend to the upper and lower quartiles within the 1.5 IQR.

To reduce stress related to cage changes and enable acclimatization to the experimenter, each individual was handled with a tunnel on four to five consecutive days before the fear conditioning experiment [36]. In addition, mice were restrained during the last two to three days of the handling procedure to familiarize them with the i.p. injections. The fear conditioning apparatus followed the design and specifications as previously described by Süß et al. [32]. On day 1 of the fear conditioning and extinction protocol, mice were placed into context A characterized by white walls, an electrified grid floor, white LED illumination (250 lx), 30% fan intensity, and 70% (v/v) ethanol as a background odor, for a 10 min habituation without stimulus presentation (Figs. 1A, 2A). The apparatus was cleaned with soap water between subjects. Fear conditioning occurred in context A on day 2. After a 2 min baseline period (Bl), mice were given five pairings of a 30 s tone (conditioned stimulus, CS, 7.5 kHz, 60 dB) with a foot shock (unconditioned stimulus, US, 0.35 mA) that co-terminated with the last second of the CS. Between stimulus presentation intertrial intervals (ITIs), ranging from 30–120 s were implemented. After the final CS/US pairing, mice were maintained in the chamber for additional 60 s (post-stimulus time, PST). On day 3, fear retrieval and extinction took place in context B, consisting of a perforated floor plate, black and white striped walls, 100% red LED illumination (28 lx), 100% fan intensity and 1% (v/v) acetic acid as background odor. 40 min prior to fear retrieval and extinction, mice received an i.p. injection of CNO (1 mg/kg) or 0.3 ml 0.9% saline solution as control. Injected individuals were moved to a separate waiting cage to reduce stress for the experimental group in the home cage. After a 2 min Bl period, mice were exposed to 14 CS presentations separated by ITIs (30–120 s), followed by a 60 s PST.

### Behavioral analysis

As previously described by Süß et al. [32], a custom-made software written in MATLAB (MathWorks) was used for video recording and stimulus presentation. Subsequently, all behavioral parameters were analyzed using EthoVision XT 15 tracking software (11.5, Noldus). The following behavioral parameters were analyzed: total distance moved (in cm), maximum velocity (in cm/s), and freezing behavior (in %). Freezing behavior was defined as the absence of any movement other than respiration for more than two seconds. The percentage of freezing behavior was binned for two CS presentations (CS 1–7). The first bin (bin1) represents fear retrieval. Individuals that did not exhibit freezing behavior were excluded from data analysis.

### Immunohistochemistry and verification of virus expression

Following the fear conditioning and extinction procedure, mice were deeply anesthetized and transcardially perfused with ice-cold PBS (1x), followed by ice-cold paraformaldehyde (4% PFA in PBS (w/v), pH 7.4, Sigma Aldrich). Brains were post-fixed in 4% PFA and cryoprotected in 30% sucrose solution in PBS at 4 °C (w/v, Sigma-Aldrich). Coronal sections of 30 µm were cut with a cryostat (CM3050 S, Leica) and collected in 24-well plates filled with PBS for subsequent *free-floating* antibody staining (see also Süß et al. [32],). Non-specific binding sites were blocked in 10% normal donkey serum (NDS, v/v, Merck Millipore) in 0.3% PBS-Triton X-100 (PBS-T, v/v, Sigma Aldrich) for 1 h at RT. To label neuronal activity, free-floating brain sections were incubated overnight at 4 °C with a primary antibody against cFos (rabbit anti-cFos, 1:500; Synaptic Systems, Cat. No. 226 008) diluted in 3% normal donkey serum (NDS) in 0.3% PBS-T (phosphate-buffered saline with 0.3% Triton X-100). The following day, sections were incubated with a secondary antibody solution containing donkey anti-rabbit Cy5 (1:500; Jackson ImmunoResearch, Cat. No. 711-175-152) diluted in 3% NDS in 0.3% PBS-T for 1.5 h at room temperature. To confirm effective DREADD activation in BNST^CRF^ neurons following CNO administration, the colocalization of mCherry-positive (DREADD-expressing) cells with cFos-immunoreactive nuclei was quantified. Data from WT and 2CKO mice receiving saline treatment were pooled for analysis. Therefore, immunolabeled brain sections were imaged using a laser-scanning confocal microscope (TCS SP5II, Leica Microsystems) with a 20 × /0.7 NA objective. Z-stacks comprising 10 optical planes were acquired. Images were processed and quantified using ImageJ software. Regions of interest (ROIs) were delineated with the “Polygon Selection tool” based on anatomical landmarks and aligned to the Allen Mouse Brain Atlas. Quantification was restricted to the BNSTad, defined by the lateral ventricle and anterior commissure. A minimum of seven ROIs per animal were analyzed. Quantification of cFos-positive, mCherry-positive, and colocalized neurons was performed manually using the “Cell Counter” plugin in ImageJ. The percentage of DREADD-expressing neurons exhibiting cFos immunoreactivity was quantified. Analyses were independently conducted by two experimenters, and the final values represent the average of both assessments. To verify accurate viral targeting, overview fluorescence images were acquired using a Leica M205 FCA fluorescence microscope (Leica Microsystems) from brain sections at caudal (AP 0.00), medial (AP + 0.15), and rostral (AP + 0.30) levels of the BNST in mice that underwent fear conditioning. Images were processed and analyzed with ImageJ software [37]. The viral pattern indicated by the expression of mCherry was redrawn, slightly colored and overlaid using CorelDRAW® Graphic Suite software (Corel GmbH). Representative confocal microscopic images (20x/0.7 NA objective) of the BNSTad region from individuals subjected to fear conditioning were taken using a Leica laser-scanning confocal microscope (TCS SP5II, Leica Microsystems). Mice lacking bilateral viral expression within the BNSTad were excluded from data analysis.

### Statistics

Graphs were created using SigmaPlot 12.5 (Systat Software) and the data were analyzed utilizing IBM SPSS Statistics (Version 29.0) software. Normality was tested before each analysis (Shapiro-Wilk test). Further, homoscedasticity (Levene’s test) was assessed, while sphericity (Mauchly’s test) was verified for cases of repeated measures. A two-way analysis of variance (ANOVA) or a three-way repeated measures ANOVA (RM-ANOVA) was used to calculate statistical significance. Groups were compared with Bonferroni’s post hoc test. Data violating any assumption were analyzed using a Kruskal-Wallis test, followed by Dunn’s post hoc test. Friedman’s test was applied in cases of repeated measures. Statistical significance was determined using a critical alpha level of 0.05 (p < 0.05). All statistical information is displayed in the appendix (Supplementary Table 1), while the most relevant results are presented in the text as mean ± SEM (p value).

## Results

### Chemogenetic inhibition of BNST^CRF^ neurons impairs fear extinction in 2CKO mice

To investigate whether a subset of neurochemically distinct CRF neurons in the BNSTad promotes fear extinction in 2CKO mice, we applied cell type-specific chemogenetic inhibition of BNST^CRF^ neurons using CRF-ires-Cre/2CKO mice (referred to hereafter as 2CKO and WT mice, respectively, Fig. 1A, B). The accuracy of virus injection and the extent of viral spread were confirmed for each individual following the FC paradigm (Fig. 1C, D). Confocal images revealed hM4Di-mCherry spread (magenta) within the dBNST was predominantly restricted to the BNSTad, largely avoiding the BNSTov (Fig. 1C; BNST boundaries exemplarily outlined by dashed lines). Viral expression was also assessed across the rostral, medial, and caudal BNST regions in each mouse. Greater blue color intensity indicated stronger expression in the dorsal BNST, while reduced intensity in the ventral BNST reflected lower expression (Fig. 1D). To examine the impact of BNST inactivation on fear extinction, we chemogenetically silenced BNST^CRF^ neurons during extinction learning (Fig. 1A, B). Mice received an intraperitoneal (i.p.) injection of CNO (1 mg/kg) 40 min prior to extinction onset to activate hM4Di-mCherry. Control mice received saline (0.3 ml, i.p.) at the corresponding time point. A significant reduction in cFos expression in hM4Di-mCherry-positive neurons confirmed effective neuronal silencing compared to saline-treated controls (Fig. 1C).

Freezing behavior during CS intervals was recorded across conditioning and extinction sessions (Fig. 1E). Statistical analysis revealed no significant differences in freezing behavior during conditioning between WT and 2CKO mice in either the saline- or CNO-treated groups. During fear retrieval (Fig. 1E, bin 1; Fig. 1J), saline-treated WT mice exhibited significantly higher CS-induced freezing than 2CKO mice. Throughout extinction, WT mice continued to display elevated freezing levels compared to 2CKO mice (Fig. 1E), consistent with previously reported accelerated extinction in 2CKO mice [32]. Importantly, chemogenetic inhibition of BNST^CRF^ neurons in 2CKO mice resulted in significantly increased CS-induced freezing and impaired extinction compared to their saline-treated counterparts (Fig. 1E). Following BNST^CRF^ neuron inactivation, freezing behavior in 2CKO mice no longer differed significantly from that of saline-treated WT mice. To rule out potential confounds related to locomotion, we analyzed behavior during the habituation phase. No significant differences were found in overall locomotor activity (Fig. 1F), indicating that 2CKO mice did not exhibit hyperactivity. Maximal movement velocity during CS/US pairings was also assessed as an index of reactivity to the unconditioned stimulus (US) (Fig. 1G) [38]. As expected, all groups showed increased velocity during CS/US trials relative to baseline (Bl). However, in contrast to previous findings, no enhanced responses were observed in either 2CKO groups. We further assessed total distance traveled and maximal velocity during the Bl period of the extinction session to evaluate any nonspecific effects of CNO or saline on motor behavior (Fig. 1H, I). Significant changes in motor behavior brought on by CNO/saline injection per se were not detected.

Taken together, our behavioral findings suggest that silencing CRF neurons in the BNSTad region enhances CS-induced fear recall and delays extinction in 2CKO mice. This supports the conclusion that a subset of GABAergic CRF neurons in the BNSTad plays a critical role in the expression of the accelerated fear extinction phenotype observed in 2CKO mice. Conversely, the activation of CRF neurons in 2CKO mice contributes to accelerated fear extinction.

### Chemogenetic activation of BNST^CRF^ neurons accelerates fear extinction in wild-type mice

Since chemogenetic inactivation of CRF neurons enhanced fear recall in 2CKO mice and impaired extinction learning, we hypothesized that chemogenetic activation of these neurons in WT animals would produce the opposite effect. To test this, 2CKO and WT mice were bilaterally injected with a double-floxed, excitatory DREADD construct (hM3Dq-mCherry; Fig. 2A, B). Mice were then subjected to the same auditory fear conditioning and extinction paradigm, with hM3Dq activation achieved via intraperitoneal CNO injection (1 mg/kg) administered 40 min before the extinction session on day 3 (Fig. 2A). Freezing behavior was recorded during the baseline (Bl) and during CS time bins across conditioning and extinction (Fig. 2E). Virus spreading was validated in all animals. The viral construct was injected into the BNSTad, however hM3Dq virus expression was also observed in the ventral BNST (vBNST) across the rostral, middle, and caudal regions (Fig. 2D). CNO-induced activation of hM3Dq-mCherry-expressing neurons in the BNSTad was confirmed by a significant increase in colocalized cFos-positive cells compared to saline-treated controls (Fig. 2C). During fear conditioning, no significant differences in freezing behavior were observed between groups (Fig. 2E). However, during extinction learning, we observed substantial genotype and treatment effects. Initially, WT mice exhibited considerably higher Bl freezing compared to 2CKO animals in the saline-treated control group. DREADD activation via CNO significantly reduced Bl freezing in WT mice to levels comparable to those of 2CKO animals. Additionally, statistical analysis revealed genotype and treatment effects in the subsequent extinction learning process. In the saline-treated hM3Dq-expressing control group, 2CKO and WT mice exhibited notable differences in freezing behavior. In contrast to the first experiment (with hM4Di), no significant group differences were observed during CS-induced fear retrieval (Fig. 2E, J). Nonetheless, WT mice exhibited persistently elevated freezing levels with a slower decline over time compared to 2CKO mice (Fig. 2E). As in the previous experiment, virus injection and experimental handling had minimal influence on baseline genotype-related differences in freezing. Again, no obvious genotype effects were detected for the total distance moved during the habituation session and maximal movement velocity during conditioning (Fig. 2F, G). Consequently, all groups displayed comparable locomotor activity and shock responsivity. However, during the Bl of the fear extinction session, significant genotype differences in locomotor activity were observed between WT and 2CKO mice, with the 2CKO control group exhibiting an increased total distance moved (Fig. 2H). Further, the maximal movement velocity was significantly increased in 2CKO mice in the initial Bl of extinction learning (Fig. 2I). Importantly, CNO treatment did not significantly affect these locomotor parameters in WT mice, indicating that the behavioral changes were not due to motor effects. Notably, CNO-induced DREADD activation significantly influenced the progression of fear extinction in WT animals. WT mice expressing hM3Dq exhibited a non-significant trend toward reduced fear retrieval (Fig. 2E, J) and a significantly faster decline in freezing behavior over time due to CNO activation (Fig. 2E). Further statistical analysis confirmed the absence of phenotypic differences in fear extinction between CNO-treated WT mice and saline-treated 2CKO mice, both expressing hM3Dq (Fig. 2E). As no significant locomotor effects were observed following CNO activation (Fig. 2H, I), the observed effects can be attributed to reduced fear behavior.

Collectively, these results support the involvement of BNST^CRF^ neurons in the extinction phenotype associated with 5-HT2CR knockout. Chemogenetic activation of distinctive BNST^CRF^ neurons in WT mice was sufficient to facilitate fear extinction, while chemogenetic inactivation of the same neurons in 2CKO mice impaired extinction and enhanced CS-induced fear retrieval.

## Discussion

This study is based on our previous findings that mice constitutively lacking the 5-HT2CR display enhanced fear extinction in an auditory fear conditioning paradigm [32]. However, the network and specific neuronal cell type mainly responsible for the differences in extinction learning phenotypes between 2CKO and WT animals could only be partially identified. Therefore, this study was designed to further analyze the neuronal substrate underlying the extinction-supporting phenotype of 2CKO mice. Our results demonstrate that this phenotype could be modified in two ways via chemogenetic modulation of CRF neurons in the BNST.

Given the predominance of GABAergic CRF neurons in the BNSTad and their known role in anxiety-related behavior, we targeted these cells to examine their influence on fear extinction in 2CKO mice.

While CRF neurons in the BNST have traditionally been associated with anxiogenic effects [39–42], more recent studies have revealed that BNST^CRF^ neurons also project to the VTA and LH in an anxiolytic manner [13, 24], with approximately 58% of BNST^CRF^ output neurons forming anxiolytic connections to the VTA or LH [13]. This aligns with the anxiolytic phenotype observed in 5-HT2CR KO mice, where reduced cFos expression in BNST^CRF^ neurons has been reported [34]. Marcinkiewicz et al. found that about 70% of dBNST^CRF^ neurons express 5-HT2CRs, indicating serotonin’s regulatory role. Our results revealed that in 2CKO mice cFos levels are increased in an extinction-supporting direction in the BNSTad [32]. Accordingly, we anticipated that the absence of the 5-HT2CR on local CRF neurons could impair the inhibition of anxiolytic projection neurons as proposed by the model of Marcinkiewicz et al., thereby accelerating extinction [13]. Additionally, we proposed that chemogenetic inactivation of CRF neurons might counteract the disinhibition caused by the absence of the 5-HT2CR in knockout mice. Indeed, we demonstrated that chemogenetic inactivation of BNST^CRF^ neurons during extinction learning enhances fear recall and impairs extinction learning in 2CKO mice. These findings are consistent with reports suggesting that a subset of CRF-expressing, GABAergic projection neurons enhances dopamine release in the VTA and exerts anxiolytic effects [12, 24]. The same study also reported that mice with chronic CRH deficiency exhibited elevated anxiety levels and increased freezing behavior in both cued and contextual fear conditioning paradigms [12]. Other studies examining the impact of biochemically distinct neuron populations in the BNST on fear behavior have yielded somewhat contradictory results. In a cued fear conditioning paradigm, Bruzsik et al. reported that neither chemogenetic inhibition nor activation of BNST^CRF^ neurons altered contextual or CS-induced fear recalls [11]. The differing results may be due to the varying experimental timelines. While Bruzsik et al. modulated CRF neurons chemogenetically during conditioning and consolidation, our modulation occurred 24 h after consolidation, just before extinction learning. Interestingly, ectopic excitation of CRF^+^ neurons in the CeA impairs fear memory acquisition and facilitates extinction, whereas CRF^+^ neuron inhibition impairs extinction memory [43]. While these findings are specific to CeA^CRF^ neurons, they point to a possible involvement of BNST^CRF^ signaling in fear extinction processes.

Consistent with the results in 2CKO mice, DREADD-dependent CNO activation of BNST^CRF^ neurons in WT mice led to a faster fear extinction, as indicated by an accelerated decline in the freezing response. Previous studies have shown that stimulation of CRF neurons in the anterior BNST is crucial for fear learning, particularly in relation to prolonged fear responses and contextual fear conditioning [13, 14]. At first glance it may seem surprising for BNST^CRF^ stimulation to decrease fear responses, given that the intra-BNST administration of CRF typically enhances both fear and anxiety by modifying neuronal circuits involved in continuous threat monitoring and stress-induced behavioral alterations [16, 44]. Importantly, the specific outcomes can vary depending on the subregion stimulated, the behavioral paradigm used, and the functional subset of neurons involved [11, 32]. According to the proposed microcircuit by Marcinkiewicz et al., the BNST contains distinct populations of CRF neurons [13]. Given that the chemogenetic method used in this study activated all CRF neurons in the BNSTad without distinguishing between interneurons and projection neurons, the overall excitation could produce a net anxiolytic effect. This is likely because the anxiolytic impact of the projection neurons probably outweighs the inhibitory effects of the interneurons. Consistent with these findings, impaired fear extinction observed in WT saline mice was ameliorated by chemogenetic activation of BNST^CRF^ neurons using hM3Dq. In addition, optogenetic activation of BNST VTA-projecting GABA neurons resulted in an anxiolytic behavioral phenotype [24]. Another study, focusing on corticotropin-releasing factor receptor type 2 (CRFR2) neurons in the posterior BNST (pBNST) demonstrated that optogenetic stimulation of these neurons could reduce anxiety, attenuate the stress response, and improve stress-induced anxiety [45]. It is important to note that the BNST is a complex structure composed of multiple functional subregions, each of which can affect anxiety-related behaviors in different ways [21]. In this context, the observed behavioral changes cannot be solely attributed to the chemogenetic modulation of CRF neurons in the BNSTad, as increased viral expression was also detected in the vBNST and juxtacellular BNST (BNSTjc). The vBNST is known to be a highly heterogeneous structure [27] that innervates the VTA [24, 46]. Neurons in the vBNST exhibit varied responses to aversive stimuli, with activation of glutamatergic vBNST projection neurons leading to aversive and anxiogenic reactions, while activation of GABAergic projection neurons tends to produce anxiolytic effects [24]. Additionally, GABAergic CRF neurons of the BNSTjc have strong connections to the CeA and project to the LH and CRF neurons of the ovBNST [44, 47]. Chemogenetic modulation of CRF projection neurons in the BNSTjc could also affect CRF neurons of the BNSTov. Therefore, the modulation of CRF neurons in the BNST and the resulting behavioral changes are not limited to the BNSTad, but may also involve the vBNST, BNSTjc, and, secondarily, the BNSTov. Nonetheless, the most significant viral expression was observed in the BNSTad.

Beyond the observed differences in the fear extinction phenotype, we identified another notable distinction between WT and 2CKO mice. Specifically, WT mice demonstrated heightened fear generalization in a safe context relative to 2CKO mice, as evidenced by the occurrence of increased Bl freezing behavior on day 2. While the genotype effect achieved statistical significance only in the hM3Dq-expressing cohort, CNO-induced DREADD activation of BNST^CRF^ neurons significantly diminished fear generalization, indicated by reduced Bl freezing in WT mice. These results are in accordance with other studies highlighting the contribution of BNST^CRF^ neurons in sustained fear, which can lead to fear generalization [16]. Interestingly, CRF knockdown in the BNST can promote fear generalization, especially following partially reinforced fear conditioning in females [16]. Our results indicate that the 5-HT2CR in the BNST must be partially involved in fear generalization, as the absence of this receptor dampens fear generalization to ambiguous cues.

The examination of certain locomotor parameters revealed partially significant differences between WT and 2CKO mice. However, these differences lacked consistency across cohorts and deviated from previous observations [32]. Earlier studies suggest that results regarding locomotor effects due to 5-HT2CR knockout are often variable and highly experiment-dependent [48]. Consistently, no significant differences were detected between treatment groups in the analyzed parameters during habituation and conditioning. This suggests an absence of pre-existing heterogeneity among groups prior to DREADD activation. Minor alterations in locomotor behavior compared to previous studies may be partially attributed to the incorporation of the CRF-ires-Cre mouse line, although this cannot be fully excluded as a contributing factor. Given the variability observed across mouse cohorts, these findings should be interpreted with caution, and additional experiments will be necessary to minimize potential confounding effects arising from behavioral variability.

In summary, serotonin in the BNST plays a complex role in anxiety modulation, primarily through its actions on different receptor subtypes. The balance between activation of anxiolytic (e.g., 5-HT1A) and anxiogenic (e.g., 5-HT2C) receptors, which are located on functionally distinct subsets of BNST^CRF^ neurons, appears to be significant for the predominant effect on anxiety-like behaviors [13, 29, 49]. Pharmacological studies have shown that 5-HT2CRs in the BNST are crucial in mediating the anxiogenic effects of acute SSRI treatment [50, 51]. 5-HT2CR activation in turn induces aversive behavior by activating BNST^CRF^ neurons, which inhibit presumed GABAergic (anxiolytic) outputs from the BNST to the VTA and LH. This effect can be blocked by 5-HT2CR antagonism [13, 51]. The absence of 5-HT2CRs on local BNST^CRF^ neurons redirects 5-HT action towards 5-HT1ARs, resulting in local disinhibition of anxiolytic BNST^CRF^ VTA/LH-projecting neurons. Consequently, in 2CKO mice, neural activity in the BNST is shifted towards anxiolytic VTA/LH projections supporting accelerated extinction learning [32]. By employing chemogenetic modulation of BNST^CRF^ neurons in both 2CKO and WT mice, we were able to alter the fear extinction phenotype bidirectionally, thus supporting this hypothesis. Our results indicate that understanding serotonin’s role in the BNST could lead to new therapeutic approaches for stress- and anxiety-related disorders. In this context, the 2CKO mice could serve as an important preclinical model for further exploration of the underlying neural mechanisms.

## Supplementary information

Suppl. Statistics Table

## Data Availability

All data supporting the findings of this study are included in the main text and its Supplementary Information files. Additional source data not included in the Supplementary Information are available from the corresponding author upon reasonable request. There are no special restrictions on access, and data will be provided without undue delay.
