# Supplementary material for: Chemogenetic modulation of CRF neurons in the BNST compensates for phenotypic behavioral differences in fear extinction learning of 5-HT2C receptor mutant mice
Source: Transl Psychiatry. 2026 Jan 10;16:63. doi: 10.1038/s41398-025-03799-1 (PMC12873198; doi:10.1038/s41398-025-03799-1)
Supplement: Supplementary file 1 — Suppl. Statistics Table [file 41398_2025_3799_MOESM1_ESM.pdf]

Figure	Statistical Test	Independent Variable	Dependent Variable		n	F/H/X^2-Statistic	df	p-value	Group Comparison	z-value	p-value
**1C**	Kruskal-Wallis Test	Treatment	Colocalization of mCherry+ and cFos+ neurons		87	48.13	1	**< *.001***	-	-	-
**1E**	Kruskal-Wallis Test	Group Affiliation	Freezing (Conditioning)	BL	24	2.11	2	.348	-	-	-
				Bin1	24	1.91	2	.384	-	-	-
				Bin2	24	1.02	2	.600	-	-	-
	Kruskal-Wallis Test & Dunn's post-hoc Test	Group Affiliation	Freezing (Extinction)	BL	24	5.90	2	.052	-	-	-
				Bin1	24	7.78	2	***.020***	KO Saline vs. KO CNO	-2.59	***.010***
									WT Saline vs. KO Saline	2.29	***.022***
				Bin2	24	12.89	2	***.002***	KO Saline vs. KO CNO	-3.06	***.002***
									WT Saline vs. KO Saline	3.25	***.001***
				Bin3	24	7.18	2	***.028***	KO Saline vs. KO CNO	-2.61	***.009***
									WT Saline vs. KO Saline	1.95	.051
				Bin4	24	7.29	2	***.026***	KO Saline vs. KO CNO	-2.31	***.021***
									WT Saline vs. KO Saline	2.44	***.015***
				Bin5	24	9.30	2	***.010***	KO Saline vs. KO CNO	-2.64	***.008***
									WT Saline vs. KO Saline	2.72	***.007***
				Bin6	24	4.66	2	.097	-	-	-

				Bin7	24	7.16	2	***.028***	KO Saline vs. KO CNO	-1.47	.143
									WT Saline vs. KO Saline	2.68	***.007***
	RM-ANOVA	Time	Freezing (Extinction): Bin1 - Bin7	KO CNO	8	9.77	6	**< *.001***	-	-	-
	Friedman's Test	Time	Freezing (Extinction): Bin1 - Bin7	KO Saline	7	22.58	6	**< *.001***	-	-	-
	RM-ANOVA	Time	Freezing (Extinction): Bin1 - Bin7	WT Saline	9	5.32	6	**< *.001***	-	-	-
**1F**	Kruskal-Wallis Test	Group Affiliation	Total Distance moved (Habituation)		24	0.57	2	.754	-	-	-
**1G**	Kruskal-Wallis Test	Group Affiliation	Maximum Velocity	BL	24	0.25	2	.987	-	-	-
				CS1 to CS5	24	1.70	2	.428	-	-	-
	Friedman's Test	Time	Maximum Velocity	KO CNO	8	8.00	1	***.005***	-	-	-
				KO Saline	7	7.00	1	***.008***	-	-	-
				WT Saline	9	9.00	1	***.003***	-	-	-
**1H**	Two-way ANOVA	Genotype	Total Distance moved (Extinction)		24	0.02	1, 21	.883	-	-	-
		Treatment			24	0.43	1, 21	.518	-	-	-
		GenotypeXTreatment			24	-	0, 21	-	-	-	-
**1I**		Genotype	Maximum Velocity		24	0.05	1, 21	.823	-	-	-

	Two-way ANOVA	Treatment	(Extinction)		24	1.58	1, 21	.223	-	-	-
		GenotypeXTreatment			24	-	0, 21	-	-	-	-
1J	Two-way ANOVA	Genotype	Freezing (Extinction)		24	7.41	1, 21	***.013***	-	-	-
		Treatment			24	10.09	1, 21	***.005***	-	-	-
		GenotypeXTreatment			24	-	0, 21	-	-	-	-
2C	Kruskal-Wallis Test	Treatment	Colocalization of mCherry+ and cFos+ neurons		71	48.01	1	< ***.001***	-	-	-
2E	Kruskal-Wallis Test	Group Affiliation	Freezing (Conditioning)	BL	22	3.30	2	.193	-	-	-
				Bin1	22	0.03	2	.984	-	-	-
				Bin2	22	1.68	2	.431	-	-	-
	Kruskal-Wallis Test & Dunn's post-hoc Test	Group Affiliation	Freezing (Extinction)	BL	22	10.15	2	***.006***	WT Saline vs. WT CNO	1.99	***.047***
									WT Saline vs. KO Saline	3.13	***.002***
				Bin1	22	7.27	2	***.026***	WT Saline vs. WT CNO	1.53	.126
									WT Saline vs. KO Saline	2.68	***.007***
				Bin2	22	9.22	2	***.010***	WT Saline vs. WT CNO	1.86	.062
									WT Saline vs. KO Saline	2.99	***.003***
				Bin3	22	10.41	2	***.005***	WT Saline vs. WT CNO	2.12	***.034***
									WT Saline vs. KO Saline	3.14	***.002***
				Bin4	22	11.76	2	***.003***	WT Saline vs. WT CNO	3.09	***.002***
									WT Saline vs. KO Saline	2.75	***.006***
				Bin5	22	8.36	2	***.015***	WT Saline vs. WT CNO	1.56	.118

									WT Saline vs. KO Saline	2.88	***.004***
				Bin6	22	5.36	2	.068	-	-	-
				Bin7	22	10.36	2	***.006***	WT Saline vs. WT CNO	2.88	***.004***
									WT Saline vs. KO Saline	2.62	***.009***
	Friedman's Test	Time	Freezing (Extinction): Bin1 – Bin7	WT CNO	7	24.67	6	***< .001***	-	-	-
				WT Saline	8	18.21	6	***.006***	-	-	-
				KO Saline	7	14.08	6	***.029***	-	-	-
**2F**	Two-way ANOVA	Genotype	Total Distance moved (Habituation)		22	1.01	1, 19	.328	-	-	-
		Treatment			22	0.01	1, 19	.931	-	-	-
		GenotypeXTreatment			22	-	0, 19	-	-	-	-
**2G**	Kruskal-Wallis Test	Group Affiliation	Maximum Velocity	BL	22	0.75	2	.687	-	-	-
				Cs1 to CS5	22	0.10	2	.950	-	-	-
	Friedman's Test	Time	Maximum Velocity	WT CNO	7	7.00	1	***.008***	-	-	-
				WT Saline	8	4.50	1	***.034***	-	-	-
				KO Saline	7	7.00	1	***.008***	-	-	-
**2H**	Two-way ANOVA	Genotype	Total Distance moved (Extinction)		22	12.38	1, 19	***.002***	-	-	-
		Treatment			22	0.74	1, 19	.400	-	-	-
		GenotypeXTreatment			22	-	0, 19	-	-	-	-
**2I**	Two-way ANOVA	Genotype	Maximum Velocity (Extinction)		22	7.57	1, 19	***.013***	-	-	-
		Treatment			22	0.06	1, 19	.814	-	-	-

		GenotypeXTreatment		22	-	0, 19	-	-	-	-
**2J**	Two-way ANOVA	Genotype	Freezing (Extinction)	22	10.36	1, 19	***.005***	-	-	-
		Treatment		22	3.97	1, 19	.061	-	-	-
		GenotypeXTreatment		22	-	0, 19	-	-	-	-
